# Architecture of the flexible tail tube of bacteriophage SPP1

**DOI:** 10.1038/s41467-020-19611-1

**Published:** 2020-11-13

**Authors:** Maximilian Zinke, Katrin A. A. Sachowsky, Carl Öster, Sophie Zinn-Justin, Raimond Ravelli, Gunnar F. Schröder, Michael Habeck, Adam Lange

**Affiliations:** 1 Department of Molecular Biophysics, Leibniz-Forschungsinstitut für Molekulare Pharmakologie (FMP), Berlin, Germany; 2 Institute of Biological Information Processing (IBI-7: Structural Biochemistry), Forschungszentrum Jülich, Jülich, Germany; 3 Jülich Centre for Structural Biology (JuStruct), Forschungszentrum Jülich, Jülich, Germany; 4 Institute for Integrative Biology of the Cell (I2BC), CEA, CNRS, Université Paris-Sud, Université Paris-Saclay, Gif-sur-Yvette Cedex, France; 5 The Maastricht Multimodal Molecular Imaging Institute (M4I), Division of Nanoscopy, Maastricht University, Maastricht, Netherlands; 6 Physics Department, Heinrich Heine University Düsseldorf, Düsseldorf, Germany; 7 AG Mikroskopische Bildanalyse, Universitätsklinikum Jena, Jena, Germany; 8 Institut für Biologie, Humboldt-Universität zu Berlin, Berlin, Germany

**Keywords:** Bacteriophages, Cryoelectron microscopy, Solid-state NMR

## Abstract

Bacteriophage SPP1 is a double-stranded DNA virus of the *Siphoviridae* family that infects the bacterium *Bacillus subtilis*. This family of phages features a long, flexible, non-contractile tail that has been difficult to characterize structurally. Here, we present the atomic structure of the tail tube of phage SPP1. Our hybrid structure is based on the integration of structural restraints from solid-state nuclear magnetic resonance (NMR) and a density map from cryo-EM. We show that the tail tube protein gp17.1 organizes into hexameric rings that are stacked by flexible linker domains and, thus, form a hollow flexible tube with a negatively charged lumen suitable for the transport of DNA. Additionally, we assess the dynamics of the system by combining relaxation measurements with variances in density maps.

## Abstract (web summary)

Bacteriophages of the *Siphoviridae* family have a long, flexible, non-contractile tail that has been difficult to characterize structurally. Here, the authors present the atomic structure of the tail tube of one of these phages, showing a hollow flexible tube formed by hexameric rings stacked by flexible linkers.

## Introduction

Tailed bacteriophages—order *Caudovirales*—comprise the prevailing majority of known phages and are subdivided into three families based on their tail morphology. *Podoviridae* feature a short tail, *Myoviridae* a long, contractile tail and *Siphoviridae* a long noncontractile, flexible tail, respectively^1^. The latter two possess a helical tail tube assembled around a tape measure protein that is tapered by a tail completion protein. Contractile tail tubes are furthermore environed by a sheath. These tail-structures are crucial for host-cell recognition, membrane penetration, and DNA transport into the host. Recently, a high-resolution cryo-EM structure of the short fiber-less tail from the *Podoviridae* T7 phage was reported at a resolution of 3.3 Å^2^. Also, a cryo-EM structure of the prehost attachment baseplate including two rings of the tail tube and sheath proteins from the *Myoviridae* T4 phage was solved at a resolution of 3.8–4.1 Å^3^, and a cryo-EM reconstruction focused solely on the tail tube was obtained from the same images at a resolution of 3.4 Å^4^. Structural analysis of the tube arrangement of *Siphoviridae* phages was for long hindered by the variable tail bending that results from its flexibility (see Fig. 1 of Tavares et al.^5^). Structural information was limited to pseudo-atomic models, which were generated for SPP1^6^ based on solution nuclear magnetic resonance (NMR) structures of monomeric tail tube proteins (TTPs), and for phages T5^7^ and λ^8^ by fitting structures of monomeric TTPs^9^ into a 6 Å cryo-EM density map. In 2020, a cryo-EM model of the baseplate of the *Staphylococcus aureus* 80α phage was reported, which includes two rings of the tail tube that are anchored within the baseplate and are, thus, not part of the flexible tube region^10^. Also, cryo-EM models of the tails of the flagellotropic tailed bacteriophage YSD1^11^ and the *Siphoviridae*-like gene transfer agent of *Rhodobacter capsulatus*^12^ were reported recently. All models show a striking structural homology between TTPs from *Myoviridae* and *Siphoviridae*; as well as between these phage TTPs and the tube-forming proteins from other injection systems, like the bacterial type VI secretion system^13^ and the extracellular injection system from bacteria and archeae^14^. The tube-forming proteins share a common fold composed of two orthogonally packed β-sheets that hexamerize through the formation of an inner β-barrel that defines the lumen of the tube. However, variable elements such as loops, N-arms and C-arms are critical to mediate intermonomer contacts driving tube assembly in these systems, and sparse high resolution data are available on the position of these elements within the long-tailed phage tubes^6–12^.

In the *Siphoviridae* SPP1 phage, the tail tube consists of the TTPs gp17.1 and gp17.1* in a ratio of 3:1, with the latter being generated by a translational frameshift adding a fibronectin type III (FN3) domain to the C-terminus of the protein. However, virions only containing gp17.1 are still viable and infectious, indicating that the additional C-terminal FN3 domain is dispensable for phage assembly and infection^15^. gp17.1 monomers are unstable in solution and spontaneously self-polymerize into long tubes in vitro which are indistinguishable from native tubes^6,16^. Previously, we presented the proton-detected solid-state NMR (ssNMR) assignment of deuterated, 100% back-exchanged gp17.1 tubes and deduced secondary structure information from the assigned chemical shifts, which confirmed and extended an existing homology model of a polymerized gp17.1 subunit^6,16^. Additionally, we introduced new concepts based on the use of specifically labeled isoleucine-methyl groups to simplify ssNMR data, inspired by earlier progress based on methyl-labeling in solution state NMR^17,18^. This allowed for the collection of unambiguous long-range distance restraints within and between subunits of the tail tube^19^.

However, due to the large size and inherent heterogeneity of this system the amount of data collected from these experiments does not suffice for a confident structure calculation. In this work, we set out to expand the labeling strategy for long-range distance restraints to further methyl groups, as well as to integrate the NMR data with a 3.5–6 Å cryo-EM map for hybrid structure calculation. Additionally, the dynamics of the system are assessed by combining relaxation data from ssNMR with variances in the cryo-EM map to create a model for tail tube bending. ssNMR is a powerful method to study the structure and dynamics^20^ of insoluble proteins, such as amyloid fibrils^21,22^ or supramolecular assemblies^23–26^. An integrated structure calculation approach^27^ in combination with cryo-EM has proven highly successful in previous applications by us^28^ and others^29–34^. The complementarity of ssNMR and cryo-EM can also be appreciated by work on bactofilin cytoskeletal filaments^35,36^.

## Results

### Hybrid structure calculation

To determine the structure of the tail-tube of SPP1, we performed a hybrid structure calculation using the inferential structure determination (ISD) approach^37^ integrating data from solid-state NMR and cryo-EM simultaneously. During structure calculation only the structure of a single monomer was represented and refined. The structures of the other subunits were generated by applying symmetry operators to the subunit structure. The use of an exact symmetry is justified by the NMR data that are only consistent with a highly symmetric sample. We represented two stacked rings in the structure calculation. Each ring was composed of six subunits such that interactions between twelve subunits were considered in the structure calculation.

**Fig. 1:**
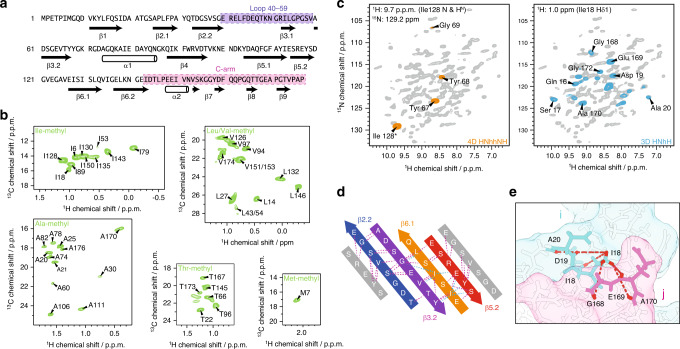
Solid-state NMR data for hybrid structure calculation defines local structure. **a** Backbone dihedral angles define the secondary structure (arrows represent β-sheets, barrels α-helices). The loop (40–59) and the C-arm (143–176) are highlighted in purple and pink, respectively. **b** Isoleucine Cδ1-methyl, alanine Cβ-methyl, leucine/valine Cδ/Cγ-methyl, threonine Cγ2-methyl, and methionine Cε-methyl labeling renders those moieties NMR-visible and yields highly resolved NMR spectra (green). **c** Long-range restraints between amide protons are extracted from a 4D HNhhNH spectrum (orange), whereas long-range restraints between amide and methyl groups are extracted from a series of 3D HNhH spectra (blue). 2D planes from both types of spectra are superimposed on a 2D hNH fingerprint spectrum (gray). **d** Representative long-range restraints (dashed lines) defining the inner β-barrel of the tail tube. The colored β-strands belong to one monomer, whereas the gray β-strands are from the neighboring subunits. Amide–amide contacts are highlighted in violet, amide–methyl contacts in cyan. **e** Schematic representation of long-range distance restraints between the Cδ1 methyl group of Ile18 and amide groups, defining the interface between the N-terminus of one monomer i (including Ile18; in cyan) and the C-arm (143–176) of another monomer j (in pink) within the tail tube. Protons are colored in red.

For the collection of solid-state NMR long-range distance restraints, we overexpressed a set of differently labeled TTP gp17.1 in *E. coli*, purified them, and let them self-polymerize into native-like tail tubes as detailed in the “Methods” section. Torsion angles were predicted based on assigned backbone chemical shifts (Fig. 1a). Specific precursor molecules were supplemented during protein expression in deuterated media, introducing NMR visible methyl groups within certain amino acids of gp17.1 (Fig. 1b). We produced samples that were homogeneously methyl and ^15^N labeled, as well as samples that were heterogeneous mixtures of 50% methyl-labeled and 50% ^15^N labeled subunits. The latter samples were used to detect intermolecular interfaces, as previously described by us^19^. All eleven investigated methyl-labeled and/or deuterated samples and their precursors are listed in Table S1. 4D and 3D proton-detected ssNMR experiments at 40 kHz magic-angle spinning (MAS) and 900 MHz proton Larmor frequency were used to probe long-range distance restraints between the following: 1) amide groups (Fig. 1c, left panel)^38,39^; 2) methyl groups; 3) methyl and amide groups globally (Fig. 1c, right panel); 4) methyl and amide groups at protein-protein interfaces. All of these experiments generated highly unambiguous restraints due to their high-dimensionality (4D) or spectral simplicity (amino-acid specific methyl labeling)—as visualized in Fig. 1d where a set of consistent restraints (amide-amide and methyl-amide contacts) defines the inner β-barrel motif of the tail tube formed by the β-strands β2.2, β3.2, β6.1, and β5.2. In mixed labeled samples (as detailed in Table S1) magnetization transfer between methyl and amide groups is solely possible at protein–protein interfaces because half of the subunits are ^15^N labeled and the other half are methyl labeled. Hence, these samples deliver a set of restraints that defines the relative organization of gp17.1 subunits within the tube. Figure 1e shows an exemplary protein interface between the N-terminus of a subunit, including the Ile18 labeled methyl group, and the C-terminus of another subunit.

**Fig. 2:**
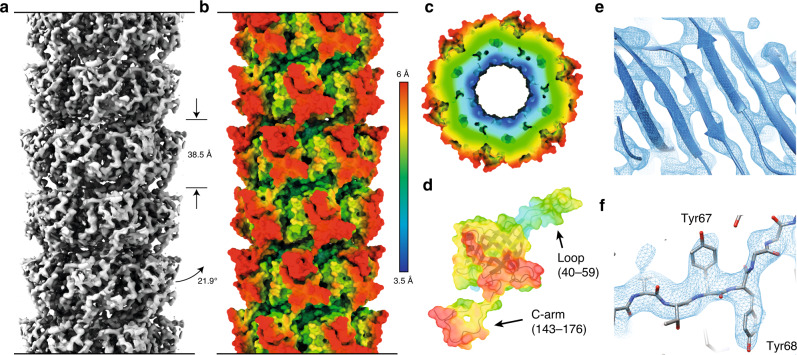
Cryo-EM data for hybrid structure calculation provides global and local information. **a** 3.5–6 Å cryo-EM map of the tail tube of SPP1 consisting of polymerized gp17.1 subunits does not only allow to deduce symmetry restraints but also limits the position of all atoms within the density. Rings with a thickness of 38.5 Å (as represented by the straight arrows) stack onto each other with a rotation of 21.9° (as represented by the bent arrow). **b**–**d** Local resolution of the cryo-EM map increases going from outer to the inner surface of the tail tube (as represented by the color gradient). **e** The inner region of the map reveals a highly resolved β-barrel and allows for the positioning of bulky sidechains as exemplified for Tyr67 and Tyr68 (**f**). The direction of the tail structure is baseplate upwards.

For cryo-EM experiments, we purified ΔN−3 gp17.1 which is indistinguishable from wt gp17.1 as judged by solid-state NMR (See Figs. S1 and S2). Curvy tubes were observed in the micrographs (Fig. S3). For image processing those tubes that appeared most straight were selected. 3D reconstruction (see “Methods” section) yielded a density map with an average resolution of 4.3 Å (Figs. 2a and S4). The local resolution varies significantly (Fig. 2b–d), from about 3.5 Å at the inner ring where the β-strands are well resolved (Fig. 2e, f), to worse than 5 Å at the periphery.

### Overall structure of the SPP1 tail tube

**Fig. 3:**
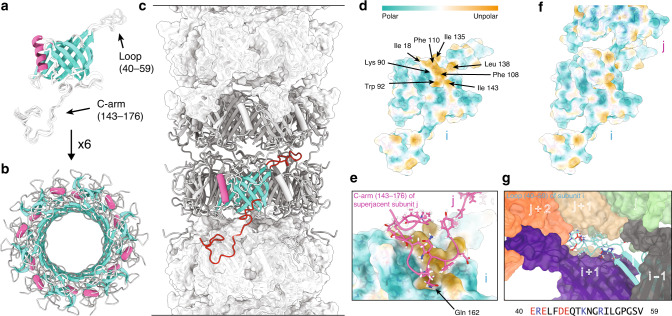
Structure of polymerized gp17.1 forming the tail tube of the bacteriophage SPP1. **a** Final ten lowest-energy structures of a gp17.1 subunit which consist of a central β-sandwich-type fold (turquoise) that is flanked by an α-helix (pink), a large loop and an extended C-terminal arm (C-arm). **b** Six gp17.1 monomers form a hexameric ring. The inner β-sheets of the β-sandwiches organize in a β-barrel motif that forms the lumen of the tube. **c** These hexameric rings stack onto each other in a helical fashion creating a hollow tube. Ring-to-ring contacts are mediated by the two loop regions (highlighted in red)—especially by the C-arm that folds onto the subjacent ring. **d** The molecular lipophilicity potential of gp17.1 reveals a hydrophobic patch on the surface of one subunit i. The color gradient represents the lipophilicity potential. **e**, **f** This unpolar area is obscured by the C-arm (pink) of the superjacent subunit j within the complex of the tail-tube—by anchoring the sidechain of Gln162 into a pocket. **g** The loop of subunit i (turquoise) features mostly electrostatic interactions with five neighboring subunits (gray, purple, green, beige, and orange) within the complex. Charged amino acids are colored in red (negative) and blue (positive). The direction of the tail structure is baseplate upwards.

Figure 3 shows the structure of the tail-tube of gp17.1 as determined by hybrid structure calculation (see also Movie S1 and Fig. S5) with a heavy atom RMSD of 1.8 ± 0.9 Å (Backbone RMSD of 1.1 ± 0.5 Å) over the entire protein sequence. The monomer of gp17.1 forms a central β-sandwich-type fold consisting of eight β-strands. This fold is flanked by an α-helix (74–86) and loop regions, of which the long C-terminal arm (C-arm, 143–176) stands out (Fig. 3a). Six gp17.1 subunits assemble into a ring with the inner 24 β-strands forming a β-barrel that defines the inner lumen of the tube. This inner lumen exhibits a negative electrostatic potential (see Fig. S6) which facilitates sliding of the viral DNA through the tube by repelling it from the surface. Additionally, inner ring contacts are mediated by the large loop region 40–59. The α-helices are arranged almost parallel to the tail tube axis (Fig. 3b). These rings stack onto each other with a rotation of 21.9° forming a right-handed helical, hollow tube (Fig. 3c). The surface of one gp17.1 subunit features a hydrophobic patch that is shaped by sidechains of various hydrophobic amino acids (Fig. 3d). In the context of the tail tube complex the C-arm of the superjacent subunit folds onto the outer β-sheet of the β-sandwich fold by anchoring the sidechain of Gln162 into a pocket (Fig. 3e). This interaction obscures the lipophilic area whereupon the complex is stabilized, because the number of unfavorable hydrophobic contacts with the solvent is reduced (Fig. 3f). This explains why a previously reported C-terminally truncated mutant of gp17.1 remains monomeric^19^. Additional ring-to-ring contacts are mediated by the loop region 40–59 which interacts with five neighboring subunits—mostly by establishing electrostatic contacts (Fig. 3g).

Structural alignments of gp17.1 and existing TTP structures of other systems show high similarity as expected (Fig. S7)—all featuring hexameric, helically stacked rings with subunits consisting of a β-sandwich-type fold and one parallel α-helix. Loop 40–59 is present at the interface between subunits in all described *Siphoviridae* phages^7,8,10,11^, *Siphoviridae*-like systems^12^ and T4 phage^4^, suggesting that it is a conserved structural element across both *Siphoviridae* and *Myoviridae* families as it was also proposed to play a regulatory role during tail polymerization. The mentioned C-arm is only present in SPP1 and 80α^10^ (even if not completely resolved). It might be a critical element regulating the tail structure in a subgroup of *Siphoviridae*. YSD1 phage features a similar intermolecular contact—an inserted domain after the α-helix that similar to the C-arm folds onto the outer β-sheet of the β-sandwich of a neighboring subunit^11^. However, this contact is established within the ring and not between the rings. Phages λ^8^ and YSD1^11^ carry an additional N-terminal loop that promotes intermolecular contacts. The organization of the tail tube of T5 phage is different since it uncommonly exhibits a trimeric ring resulting from the fusion of every two subunits within the hexamerization domain^7^. The *Siphoviridae*-like gene transfer agent tail tube does not bear any of these elements^12^. The *Myoviridae* T4^4^ phage TTP gp19 features two additional linkers that mediate intermolecular interactions—which facilitates contact to ten different subunits over an area of 6706 Å^2^ within the tail tube. gp17.1, however, only interconnects with six different subunits over 4850 Å^2^. This dramatically reduced contact—in addition to not being bundled in a sheath—is expected to enable flexibility of this *Siphoviridae* tail tube as proposed previously for phages T5 and λ^7,8^.

### Dynamic regions mediate tail bending

**Fig. 4:**
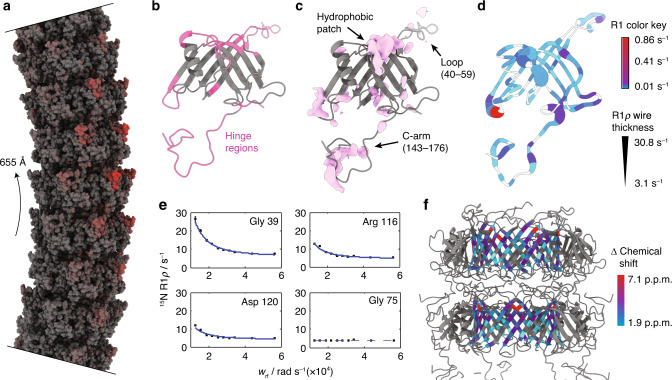
Bending of the tail tube is mediated by flexible hinge regions. **a** Model of a bent SPP1 tail tube with a curvature radius of 655 Å (as indicated by the arrow). The model is based on the structure of straight tubes and 2D class averages of bent tubes. Most structural changes are found on the outside of the curve (red) which implies that the bending process is mediated by stretching. **b** Regions that act as hinges during bending of the tube are colored in pink. **c** Variances in the cryo-EM map (pink) match the hinge regions. **d** Also, hinge regions are associated with highest ^15^N R_1_ (color key) and R_1ρ_ (thickness of wire) relaxation rates. White coloring represents missing values. **e** Decaying relaxation dispersion profiles indicate the presence of slow motions. **f** Relaxation dispersion profiles of the inner β-barrel can be fitted in a correlated manner to a two-state exchange process. Smallest chemical shift changes correlate with middle regions of the β-barrel which are furthest away from the hinge regions (color key). The direction of the tail structure is baseplate upwards. Source data are provided as a Source Data file.

To determine the driving forces contributing to the flexibility of the tail tube of SPP1, we created a model of a bent tail tube based on the structure of monomeric gp17.1 and 2D class averages of bent tubes as detailed in the “Methods” section. As shown in Fig. 4a (Movie S2), most structural changes during the bending process happen on the outer edge of the curve while the inside of the curve remains unchanged compared to the straight filament. This suggests that the bending of the tube is facilitated by stretching of certain linker regions (as opposed to compression). The hinge regions required for the structural reorganization from a straight to a bent state (Fig. 4b) match regions in the cryo-EM density with pronounced variances (Fig. 4c). These areas comprise regions forming inter-ring contacts—especially the C-arm (143–176), its binding interface on the subjacent subunit and the loop (40–59).

Additionally, we analyzed ^15^N R_1_ and ^15^N R_1ρ_ relaxation rates of fully polymerized gp17.1 by solid-state NMR as detailed in the “Methods” section (Fig. S8). R_1_ relaxation rates are sensitive to motions on the nanosecond timescale, whereas R_1ρ_ rates report on motions on the nanosecond to millisecond timescale^40^. Figure 4d shows both values mapped onto a gp17.1 subunit within the tail complex. The inner β-barrel shows nearly no motion on these timescales—which correlates with these areas being highest resolved in the cryo-EM density. On the opposite, the hinge regions are associated with the highest relaxation rates, demonstrating that these areas are highly dynamic. Furthermore, we investigated ^15^N relaxation dispersion which is sensitive to motions on the millisecond timescale (Figs. S9–S11). Figure 4e shows representative ^15^N relaxation dispersion curves; flat profiles indicate the absence of motion, whereas decaying profiles indicate the presence of dynamics. Most residues are involved in slow motions (Fig. S12). The residues belonging to the β-barrel can be fitted in a combined approach to a two-state model assuming in a simplified way the existence of two distinct conformations of the β-barrel (Fig. 4f). Calculated chemical shift differences between both states are higher for residues in proximity to the hinge regions. Thus, we propose that these slow motions represent tube bending. The global, collective nature of this motion does not impose heterogeneity onto the cryo-EM map since only straight tubes are considered for structure calculation.

Overall, our hybrid data support a model where the C-arm (143–176) and the loop (40–59) act as bellows contributing to tail tube bending by stretching. Our dynamic structure of the tail tube is reminiscent of a molecular spinal column. The hexameric rings forming the inner β-barrel would be in this picture the vertebrae, while the flexible parts (C-arm and loop) correspond to the intervertebral disks. The flexibility of the system might facilitate the screening of the bacterial membrane to find the receptor for infection initiation. We expect that the combination of sophisticated ssNMR experiments and cryo-EM will help to characterize structures of other dynamic and/or flexible supramolecular assemblies, in particular those systems where the conformational flexibility leads to a lack of resolution in cryo-EM reconstructions—while depending on the timescale of these dynamics the quality of NMR spectra may not be affected.

## Methods

### Preparation of deuterated protein samples for proton-detected solid-state NMR measurements

Protein samples and their preparation are summarized in Table S1. gp17.1 protein was expressed, purified and polymerized as described in the following. *E. coli* BL21 were transformed with a pETM13 vector containing the gp17.1 sequences including a C-terminal His-tag (Fig. S1). In a three step protocol, the bacterial cultures were adapted to D_2_O conditions: In the first step, 12.5 mL LB medium was mixed with 12.5 mL fully deuterated M9-medium with ^13^C,D_7_-glucose and ^15^ND_4_Cl as the sole carbon and nitrogen sources. By lyophilizing and re-dissolving in D_2_O twice, exchangeable protons of the M9 medium components (ammonium chloride, salts, and trace elements) were replaced by deuterons. After pre-warming this LB/M9 (50%/50% H_2_O/D_2_O) mixture to 37 °C, it was inoculated with a glycerol stock, and incubated at 37 °C and 150 rpm for 4 h. In the second step, protons were further diluted by adding 25 mL fully deuterated M9-medium, followed by incubation of the (25%/75% H_2_O/D_2_O) medium for 4 h (37 °C, 150 rpm). In the last step, 200 mL prewarmed, fully deuterated M9-medium was added to the bacterial culture. The mixture was incubated at 30 °C and 150 rpm overnight (5%/95% H_2_O/D_2_O). The next morning, the D_2_O-adapted bacteria were spun down at 3500 × *g* and 37 °C for 20 min and gently resuspended in 1 L fully deuterated M9 medium to an OD of 0.1 for final expression.

The D_2_O-adapted bacterial cultures were incubated at 37 °C and 150 rpm until an OD of 0.8. In D_2_O dissolved IPTG was added to a final concentration of 1 mM to the bacterial cultures to induce protein expression for 4 h. By centrifugation for 20 min at 4650 × *g*, the bacteria were harvested. The bacteria pellets were resuspended in 50 mL lysis buffer (20 mM sodium phosphate, 8 M urea, 15 mM imidazole, 0.5 M sodium chloride, 5% glycerol (v/v), 0.1% Triton X-100 (v/v), pH 7.4). The resuspended pellets were incubated under agitation at room temperature overnight.

DNA was disrupted by sonication in order to reduce the viscosity of the lysate using a BRANSON digital sonifier (model 250D, using a micro tip with max. temperature 75 °C, 40% amplitude and 20 min sonication time). The lysate was clarified by centrifugation at 30,000 × *g* for 20 min. It was diluted to a volume of 200 mL with lysis buffer and loaded onto a pre-equilibrated (buffer A: 20 mM sodium phosphate, 8 M urea, 15 mM imidazole, 500 mM sodium chloride, pH 7.4) nickel column (5 ml HisTrap HP) using an Äkta system. The column was washed with 5 column volumes of buffer A and the protein was eluted in 5 column volumes of buffer B (20 mM sodium phosphate, 8 M Urea, 1 M imidazole, 500 mM sodium chloride, pH 7.4). The elution fractions were checked by SDS-PAGE and those containing pure gp17.1 were pooled. The protein sample was dialyzed three times (1 h, 2 h, overnight) against 1 L buffer C (20 mM sodium phosphate, 500 mM sodium chloride, and 1 mM EDTA).

After dialysis gp17.1 was left for 3 weeks at room temperature to polymerize. The gp17.1 filaments were sedimented by ultracentrifugation at 90,000 × *g* for 2 h. ~100 mg of gp17.1 protein pellet could be isolated^16^.

For methyl-labeling, ^12^C_6_,D_7_-glucose was used instead of ^13^C_6_,D_7_-glucose and specific precursor molecules were supplied to the bacterial cultures 1 h prior to induction (Table S1). For mixed-labeling, two differently labeled samples, e.g., 50% methyl-labeled and 50% ^15^N-labeled, were produced and mixed before polymerization^19^. Labile protons were 100% back-exchanged in all samples. The protein pellets, a few DSS crystals for spectral referencing and temperature control, and 1 µL of D_2_O for field locking were filled into 1.9 mm rotors provided with bottom spacers.

### Preparation of fully protonated protein samples for carbon-detected solid-state NMR and cryo-EM measurements

gp17.1 and Δ*N*−3 gp17.1 were expressed, purified, and polymerized as described above, only that D_2_O was exchanged to H_2_O. Δ*N*−3 gp17.1 for cryo-EM was expressed in LB medium. The protein pellets and a few DSS crystals for spectral referencing and temperature control were filled into 3.2 mm rotors. For cryo-EM, 20 mM sodium phosphate in the final buffer was replaced by 20 mM Tris-HCl (pH 7.4).

### Solid-state NMR spectroscopy

Solid-state NMR spectroscopy of the methyl-labeled and/or deuterated protein samples was conducted with a 1.9 mm, four-channel (^1^H, ^13^C, ^15^N, and ^2^H) probe at 40 kHz magic-angle spinning (MAS) frequency and an external magnetic field strength according to 900 MHz ^1^H Larmor frequency. The temperature was calibrated to around +18 °C by means of internally added DSS. 2D hCH and 3D HNhH spectra were recorded as described previously^19^, a 2D hNH spectrum was recorded as detailed before^16^. Pulse program, acquisition, processing and reconstruction parameters for the 2D hCH, 3D HNhH, 3D HChH, and 4D HNhhNH spectra are summarized in Supporting Information Tables S2–S6.

Solid-state NMR spectroscopy of the fully-protonated samples was conducted with a 3.2 mm, triple-channel (^1^H, ^13^C, and ^15^N) probe at 11 kHz MAS frequency and an external magnetic field strength according to 900 MHz ^1^H Larmor frequency. The temperature was calibrated to around +10 °C by means of internally added DSS. 2D ^13^C–^13^C correlation spectra with 50 ms proton-driven spin diffusion (PDSD) mixing were recorded as fingerprints to compare wild-type gp17.1 with the Δ*N*−3 gp17.1 mutant.

Long-range distance restraints were extracted from the recorded spectra by peak picking in CcpNmr^41^. Methyl groups were assigned based on our previous work^19^, on the basis of the assignment precursor (Table S1) or by correlations to sequential amide groups. In the alanine-methyl sample (Table S1) protons scrambled into the Hγ2 position of isoleucines.

### Relaxation measurements by solid-state NMR

^15^N R_1_ and ^15^N R_1ρ_ relaxation rates were measured by a series of pseudo-3D experiments (^1^H, ^15^N, delay/spinlock strength). The delay times for the R_1_ pseudo-3D experiments were 0.5, 1, 1.5, 2, 3, 4, 8, 16, and 32 s. For the R_1ρ_ pseudo-3D experiments spinlock strengths of 9, 7, 5.5, 5, 4.5, 4, 3.5, 3, 2.5, and 2 kHz and spinlock durations of 1, 5, 10, 20, 40, 80, 100, 140, and 200 ms were used. The peak heights from the resulting 2D hNH correlation spectra were extracted with CcpNmr^41^ and fitted as a function of relaxation time to a monoexponential function. This results in one global *R*_1_ relaxation rate for each—in the 2D hNH spectra distinguishable—amide nitrogen and various spinlock strength-dependent *R*_1ρ_ relaxation rates. For relaxation dispersion analysis the *R*_1ρ_ values are plotted against the spinlock strength and fitted to a two-site Bloch–McConell equation^42^:

1 $$R_{1\rho }\,=\,R_{1\rho ,0} + \frac{{p_{\mathrm{A}}p_{\mathrm{B}}{\mathrm{{\Delta} }}\delta ^2k_{{\mathrm{ex}}}}}{{\omega _1^2 + k_{{\mathrm{ex}}}^2}} = R_{1\rho ,0} + \frac{{\varphi _{{\mathrm{ex}}}k_{{\mathrm{ex}}}}}{{\omega _1^2 + k_{{\mathrm{ex}}}^2}}.$$

For all resolved residues that are part of the β-barrel forming the inner lumen of the tail tube a combined fit was conducted with a single *k*_ex_ coefficient for all residues and individual *φ*_ex_ and *R*_1ρ,0_ for each residue (See Table S7). The best fit was achieved by minimization of the target function *χ*^2^ as demonstrated previously^43^. On-resonance *R*_1ρ_ relaxation rates were deviated from the observed *R*_1ρ,obs_ relaxation rates, *R*_1_ relaxation rates and the angle between the spinlock offset frequency (*ω*_1_), and the chemical shift offset from that (Ω) in a residue specific manner:

2 $$R_{1\rho }\,=\,\frac{{R_{1\rho ,{\mathrm{obs}}}\,-\,R_1\,{\mathrm{cos}}^2\theta }}{{{\mathrm{sin}}^2\theta }},$$

3 $$\theta\,=\,{\mathrm{tan}}^{ - 1}\frac{{\omega _1}}{{\mathrm{{\Omega} }}}.$$

The errors were estimated by Monte Carlo simulations. The fits were repeated 250 times with the *R*_1ρ_ errors being multiplied by a random number between 0 and 1. The *R*_1ρ_ errors were calculated in a similar way by performing the fits 1000 times using average noise from the spectra as input.

### Cryo-EM image acquisition

Cryo-preparation was performed on glow-discharged holey carbon films (Quantifoil R 1.2/1.3, 300 mesh) using a Vitrobot (FEI). With 110,000-fold nominal magnification 855 micrographs have been recorded on a Tecnai Arctica electron microscope operating at 200 kV with a field emission gun using a Falcon III (FEI) direct electron detector in integrating mode directed by EPU data collection software (version 1.5). Each movie was composed of 20 fractions. Each fraction contained six frames, i.e., a total of 120 frames were recorded per micrograph. The sample was exposed for 3 s to a total dose of 70 e^−^/Å^2^. Applied underfocus values ranged between 0.2 and 1.7 µm. The pixel size was calibrated to 0.935 Å as calibrated using gold diffraction rings within the powerspectra of a cross grating grid (EMS, Hatfield). Details of data acquisition are summarized in Table S8.

### Cryo-EM image processing and helical reconstruction

MotionCor2^44^ was used for movie correction and CTF parameters were fitted with Gctf^45^. All image processing was done using RELION 2.1^46^. Fibrils were manually picked, and segments were extracted with an interbox distance of 10% of the box sizes, which yielded 69,282 segments. Box sizes were chosen as 200 pixels. Segments from micrographs for which Gctf estimated a resolution worse than 5 Å were discarded, which left 64,760 segments. 2D classification was used to select those classes that show sufficient detail in the class averages (Fig. S13). The initial model for 3D reconstruction was built with the Relion relion_helical_toolbox program with the –simulate helix option, which places spheres along a helix, using a rise of 40 Å and a twist of 21°.

The initial model was refined using 3D classification (*K* = 3). The class yielding the highest resolution was used as initial model for further 3D classification with all particles using *K* = 5 classes and a *T* value of 4. The best resolved class contained 10,682 segments which were used for further refinement. A soft mask enclosing three rings was used for further refinements. The number of filaments and segments used for the final reconstruction was 1866 and 5965, respectively. The final optimized helical symmetry was C6, with a helical rise of 38.46 Å and a twist of 21.89°. Gold-standard refinements were performed by selecting entire fibrils and splitting the data set accordingly into an even and odd set. The Fourier shell correlation was computed between two half maps. According to the 0.143 criterion the obtained resolution is 4.3 Å (Fig. S14). To obtain a robust resolution estimate, the FSC curve was fitted using 1/[e^((x−A)/B)^ + 1]^C^, yielding *A* = 0.122, *B* = 0.015, and *C* = 0.228. The 0.143 criterion then yields a resolution of 4.0 Å (Fig. S14). Image processing and reconstruction details can be found in Table S8. The final map was sharpened with the EMAN2 tool e2proc3d.py with a B-factor of −150 Å^2^, locally normalized and filtered to 3.5 Å.

The local resolution as shown in Fig. 2b–d, was estimated by comparing a density map computed from the atomic model with the reconstructed density map by FSC. Both density maps were first interpolated on finer grid (pixel size of 0.468 Å). The (local) FSC calculation was done with the EMAN2 program e2fsc.py, using a FSC cutoff of 0.5 for defining the resolution.

### Structure calculation

The hybrid structure calculation aims to combine the experimental data from NMR and cryo-EM. Distance restraints were derived from NMR peak lists and incorporated using a logistic restraint potential similar as described before^47^. The density map from cryo-EM was incorporated as a real-space map restraint^48^. Both data sets pose their own challenges: The NMR distance restraints are highly ambiguous due to the helical symmetry of the tail tube. NMR peaks stemming from the homogeneously mixed samples can result from a contact within the monomer or from contacts between different subunits (i.e., between the monomer and one of its virtual copies generated by the symmetry operators). However, NMR peaks stemming from the heterogeneously mixed samples can clearly be assigned as intermolecular long-range restraints. We considered all possible interactions between all members of both hexameric rings. Therefore, in total 12 possible contacts were combined as an ambiguous distance restraint. Another challenge is posed by the generous upper bound of 7 Å for the distance restraints. Also, the cryo-EM map itself has a substantial resolution inhomogeneity and is not sufficient for an unambiguous tracing of the backbone in the outer β-strands and the C-terminus.

A particular challenge was posed by the estimation of the registers of adjacent β-strands. Due to the insufficient resolution of the density map in the flexible regions (e.g., residues 40–49) and the large distance upper bounds, many relative registers between adjacent strands seem possible in principle. To infer the register that is most consistent with the NMR and cryo-EM data, we developed a new probabilistic restraint that probes all possible registers between adjacent strands. The estimated registers were then imposed as additional hydrogen bonding restraints to increase the regularity of the gp17.1 tail tube structure.

Only the combination of the local information provided by the NMR restraints with the global shape information encoded in the cryo-EM map allowed us to compute a near-atomic structure of the tail tube of gp17.1. For example, in the initial phase of the project we tried to compute a structure based only on the NMR restraints and homology information with literature values for the symmetry parameters. Although the β-sandwich and the α-helix can be computed from this information, it was not possible to model the loop (40–59) and the C-terminus correctly. Due to the ambiguity of the NMR restraints resulting from the helical symmetry it is not clear from the NMR data which subunit of the subjacent ring is contacted by the C-arm.

In the final stage of the hybrid structure calculation, we used an iterative approach in which hybrid structure calculation with ISD was alternated with a pure EM refinement using Coot^49^ and Phenix^50^. MDFF simulations for 5 ns were used on intermediate models to guide model building in Coot.

Finally, two different models were generated for final interpretation (and were also deposited to the PDB): one (ensemble of) models represents the ensemble from the final ISD refinement (PDB ID: 6YQ5). The other model (PDB ID: 6YEG) represents a standard refinement against the EM density (including cycles of Coot and Phenix) starting from the hybrid NMR–EM model obtained from ISD. The statistics of the solid-state NMR long-range distance restraints, the violations and the RMSDs of the final ensemble are summarized in Tables S9–11.

### Variance map

To determine the structural variance in the dataset a bootstrapping analysis was performed. For this, 300 density maps were reconstructed (with fixed orientations and shifts) from randomly resampled (with replacement) sets of segment images, using the relion_reconstruct command with C6 and helical symmetry.

The density variance calculated directly over such resampled density maps often leads to artifacts (strong noisy variance outside the particle and strong variance at symmetry axes). We therefore computed instead the isosurface variance map, which yields a clearer view of the structural variance. To compute the isosurface variance, all 300 density maps were first low-pass filtered, and then masks were computed using a density threshold of 0.162. Then a Gaussian filter was applied to all 300 masks. Finally, the variance of all masks was computed, which yields the isosurface variance map (Fig. 4c). Since symmetry was used during the reconstruction, symmetry-breaking variance is not visible.

### Model of a bent SPP1 tail tube

For the analysis of the curved filament regions, curved filaments were picked in short segments. The average number of segments per picked filament was only 4, whereas it was 13 for the straight filaments. 12,259 segments was obtained from the curved filaments, which were extract with a larger box size of 400 pixels to clearly see the curvature. From a 2D classification with 50 classes the best defined classes were chosen and yielded 2735 segments. Further 3D classification yielded a final reconstruction with 1,418 particles at a resolution of only about 17 Å. Due to the curvature, no helical symmetry could be used.

On the basis of the 2D class averages of bent tail tubes (Fig. S15) a curvature radius of 655 Å (inner radius = 655 − 63.3/2 = 623.4 Å; outer radius = 655 + 63.3/2 = 686.7 Å), an inner distance between subunits of 38.5 Å, an outer distance between subunits of 47.0 Å and an angle between subunits of 3.5° could be determined. The distance between neighboring rings on the inside is the same as in the straight filament, however, the distance on the outside is larger. The curvature is therefore induced by stretching the outside while the inside distances remain unchanged compared to the straight filament. The curvature from the 2D class averages represents the average, most populated curvature. A maximum curvature of 560 Å could be extracted from the micrographs which results in a maximum angle between subunits of 4.2° (Fig. S3). This geometric information was used to build a 10-ring bent tail tube from gp17.1 monomers. For this, copies of one ring from the straight tail tube were translated and rotated accordingly.

The rationale for building this bent model was to impose the observed curvature and ring distances, but to keep the local structure and subunit contacts as similar as possible to the straight tail tube, as we do not have high-resolution information on the curved tail tube. Therefore, a network of harmonic distance restraints (random atom pairs between 3 and 15 Å) was defined with target distances from the straight tail tube. In addition the β-sheets at the inside of the tubes were position-restrained to keep curvature and relative ring positions. DireX^51^ was used to optimize the model under these distance and position restraints (without density map restraints). The curved model (Fig. 4a) represents a model that is closest (in local structure and subunit contacts) to the straight tail tube, while adopting the imposed curvature and ring distances. The amount of fulfilled restraints after bending reveals regions of the protein that are exposed to environment changes (red color coding in Fig. 4a).

ChimeraX^52^ morph command was used to create a trajectory between the straight and the bent tail tube (standard settings). During that procedure hinge and core regions are identified by a reimplementation of the morph server^53^.

### Figure creation

Figures were created with ChimeraX^52^.

### Reporting summary

Further information on research design is available in the Nature Research Reporting Summary linked to this article.

## Supplementary information

Supplementary Information

Description of Additional Supplementary Files

Supplementary Movie 1

Supplementary Movie 2

Reporting Summary

## Data Availability

Solid-state NMR chemical shift assignments are deposited at the Biological Magnetic Resonance Data Bank under the accession code 27468. The cryo EM electron density map is deposited at the Electron Microscopy Data Bank under the accession code EMD-10792. Protein structures are deposited at the Protein Data Bank under the accession codes 6YEG (hybrid structure of the SPP1 tail tube by solid-state NMR and cryo EM; final EM refinement) and 6YQ5 (hybrid structure of the SPP1 tail tube by solid-state NMR and cryo EM; NMR ensemble). The authors declare that any other data supporting the findings of this study are available within the article and in its Supplementary Information, or from the authors upon request. Source data are provided with this paper.

## Source data

Source Data

## Code availability

Codes for curve fitting, error analysis, and structure calculation are available from the corresponding authors upon request.
