# Supplementary material for: Architecture of the flexible tail tube of bacteriophage SPP1
Source: Nat Commun. 2020 Nov 13;11:5759. doi: 10.1038/s41467-020-19611-1 (PMC7666168; doi:10.1038/s41467-020-19611-1)
Supplement: Supplementary file 1 — Supplementary Information [file 41467_2020_19611_MOESM1_ESM.pdf]

Supporting Information for

Architecture of the flexible tail tube of bacteriophage SPP1

Maximilian Zinke[1], Katrin A. A. Sachowsky[2,3], Carl Öster[1], Sophie Zinn-Justin[4], Raimond Ravelli[5], Gunnar F. Schröder[2,3,6], Michael Habeck[7], Adam Lange[1,8]

[1] Department of Molecular Biophysics, Leibniz-Forschungsinstitut für Molekulare Pharmakologie (FMP), Berlin, Germany

[2] Institute of Biological Information Processing (IBI-7: Structural Biochemistry), Forschungszentrum Jülich, Jülich, Germany

[3] Jülich Centre for Structural Biology (JuStruct), Forschungszentrum Jülich, Jülich, Germany

[4] Institute for Integrative Biology of the Cell (I2BC), CEA, CNRS, Université Paris-Sud, Université Paris-Saclay, Gif-sur-Yvette Cedex, France

[5] The Maastricht Multimodal Molecular Imaging Institute (M4I), Division of Nanoscopy, Maastricht University, Maastricht, Netherlands

[6] Physics Department, Heinrich Heine University Düsseldorf, Düsseldorf, Germany

[7] AG Mikroskopische Bildanalyse, Universitätsklinikum Jena, Jena, Germany

[8] Institut für Biologie, Humboldt-Universität zu Berlin, Berlin, Germany.

These authors contributed equally: Maximilian Zinke, Katrin A. A. Sachowsky. Correspondence and requests for materials should be addressed to G.F.S. (email: gu.schroeder@fz-juelich.de) or to M.H. (email: michael.habeck@med.uni-jena.de) or to A.L. (email: alange@fmp-berlin.de)

a

```
>gp17.1:
1   MGMPETPIMGQDVKYLFQSIDAATGSAPLFPAYQTDGSVSGERELFDEQT
51  KNGRILGPGSVADSGEVTYYGKRGDAGQKAIEDAYQNGKQIKFWRVDTVK
101 NENDKYDAQFGFAYIESREYSDGVEGAVEISISLQVIGELKNGEIDTLPE
151 EIVNVSKGGYDFQQPGQTTGEAPGTVPAPHHHHHH
```

b

```
>ΔN-3 gp17.1:
1   MPIMGQDVKYLFQSIDAATGSAPLFPAYQTDGSVSGERELFDEQTKNGRI
51  LGPGSVADSGEVTYYGKRGDAGQKAIEDAYQNGKQIKFWRVDTVKNENDK
101 YDAQFGFAYIESREYSDGVEGAVEISISLQVIGELKNGEIDTLPEEIVNV
151 SKGGYDFQQPGQTTGEAPGTVPAPHHHHHH
```

Figure S1. Protein sequences of gp17.1 used in this study: a) gp17.1 wt and b) ΔN-3 gp17.1

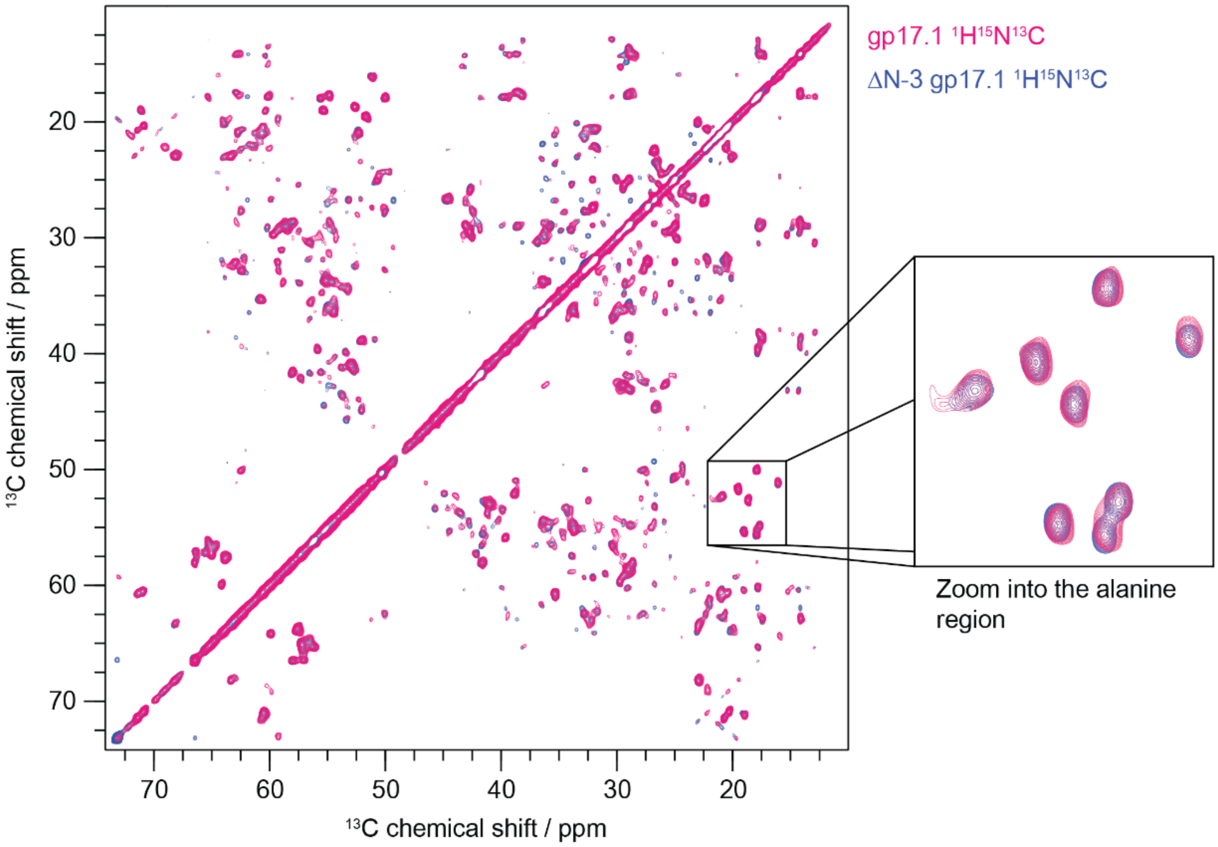

Figure S2. Aliphatic region of 2D hCC solid-state NMR correlation spectra of fully-protonated gp17.1 (pink) and ΔN-3 gp17.1 (blue) tail tubes at 11 kHz MAS and 900 MHz external magnetic field strength. The zoom into the alanine region reveals that both spectra show the same fingerprint, demonstrating that the N-terminal truncation does not impair the structural organization of the tail tube.

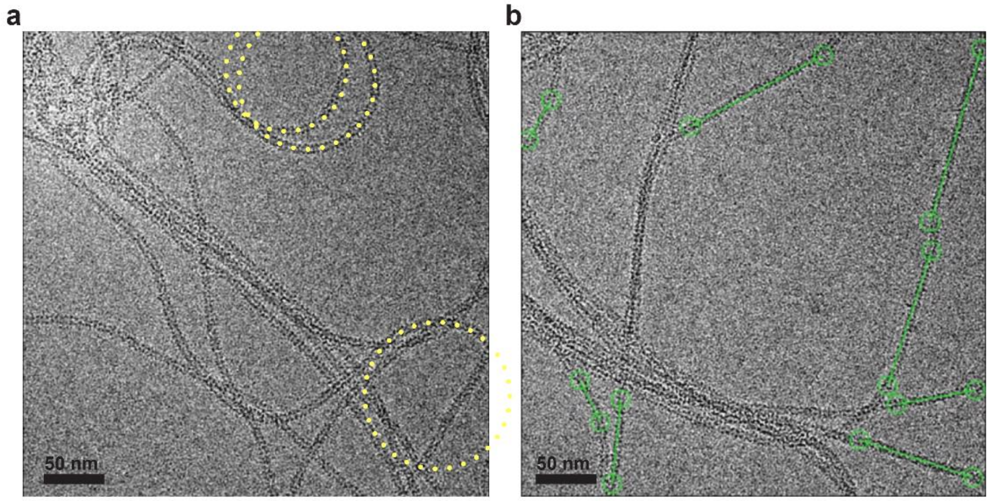

Figure S3. Typical micrographs of polymerized gp17.1 tail tubes. a) The flexible tubes show variable bending and cross each other. Yellow circles represent the maximum observed curvature of the tail tubes as described in the methods. The curvature was averaged over the five most bent tubes observed in the micrographs. b) Exemplary picked straight filament segments for cryo-EM image processing. The green circles label the starting and ending coordinates. The scale bar measures 50 nm.

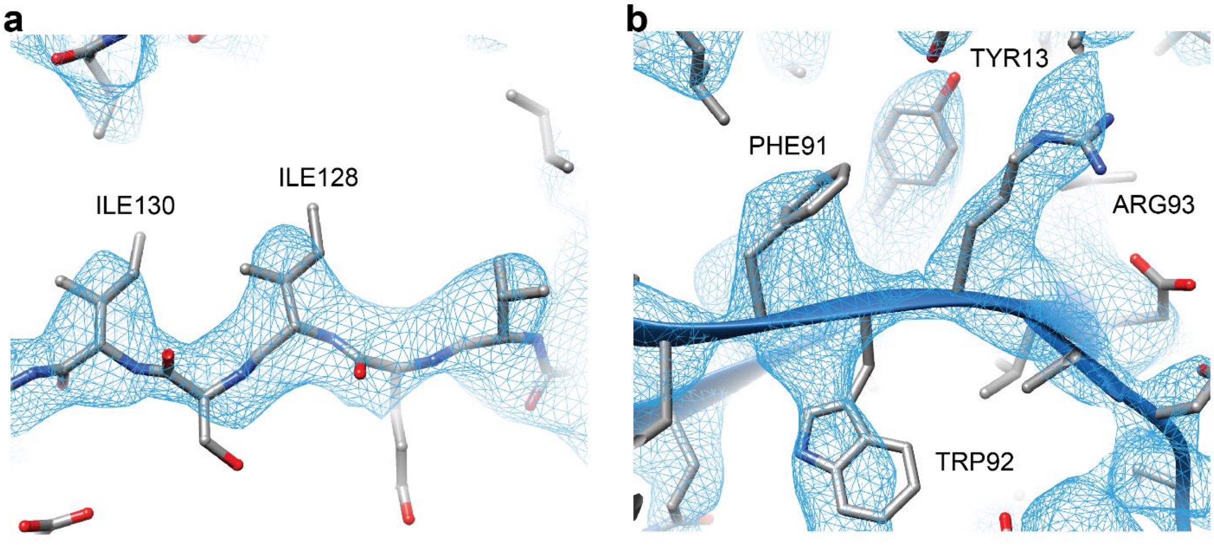

Figure S4. Quality of the cryo-EM map. Cryo-EM density of the inner (a) and outer β-sheet (b) resolves sidechains.

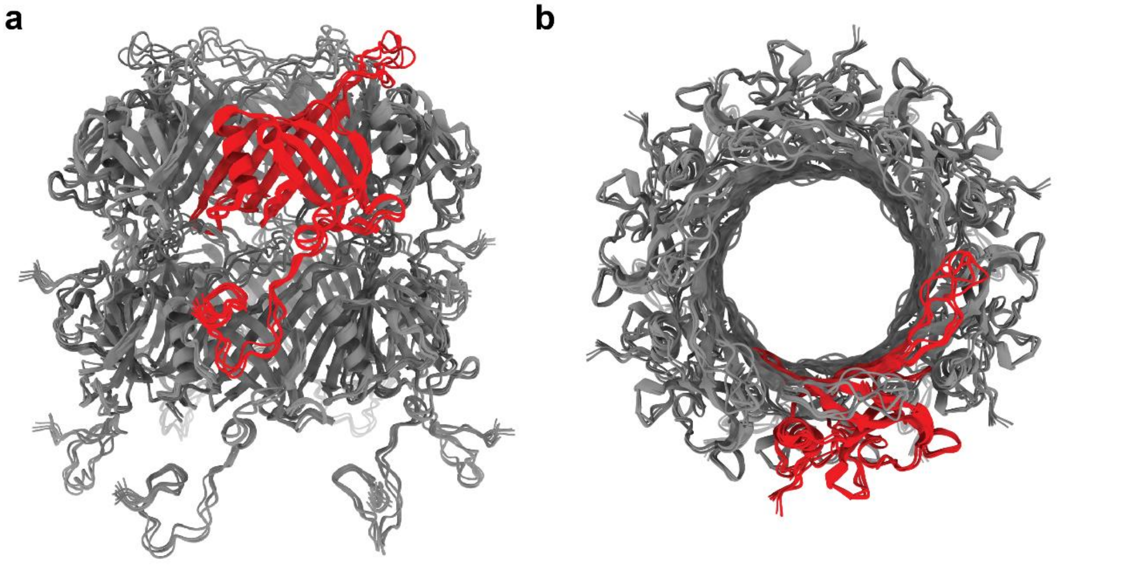

Figure S5. Final ten lowest-energy structures of two SPP1 tail-tube rings after hybrid structure calculation (PDB ID 6YQ5). One gp17.1 subunit within the top ring is highlighted in red. The assembly is shown from the side a) and from the top b). The direction of the tail structure is baseplate upwards.

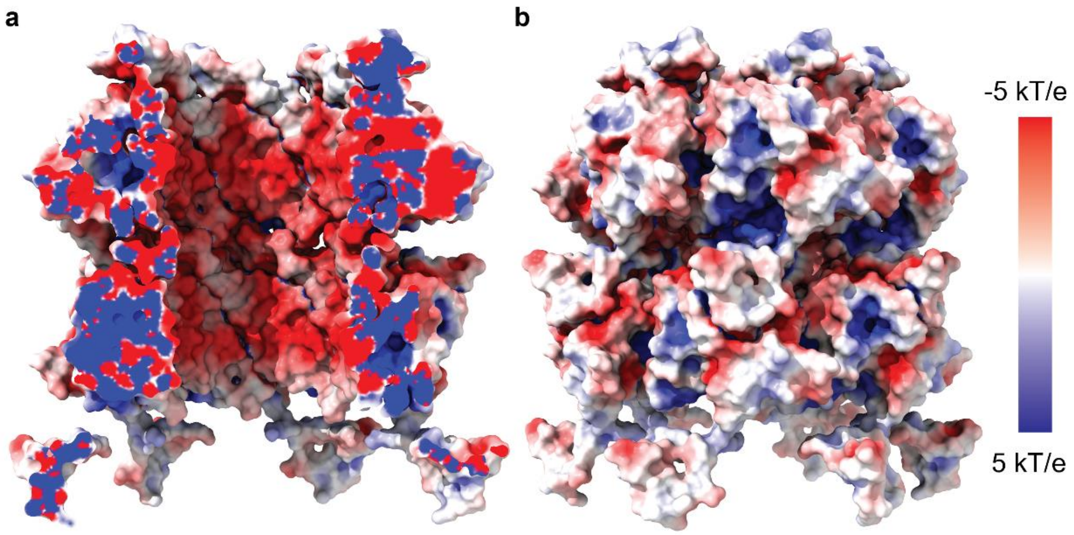

Figure S6. Electrostatic potential of two rings of the SPP1 phage tail-tube. The structure (PDB ID 6YQ5) was prepared with PDB2PQR[1] and the potential was computed with the Adaptive Poisson-Boltzmann Solver (APBS) program[2]. The color gradient represents the electrostatic potential. The tail features a highly negatively charged lumen. The tail-tube is shown in a side view from the a) inside and b) outside. The direction of the tail structure is baseplate upwards.

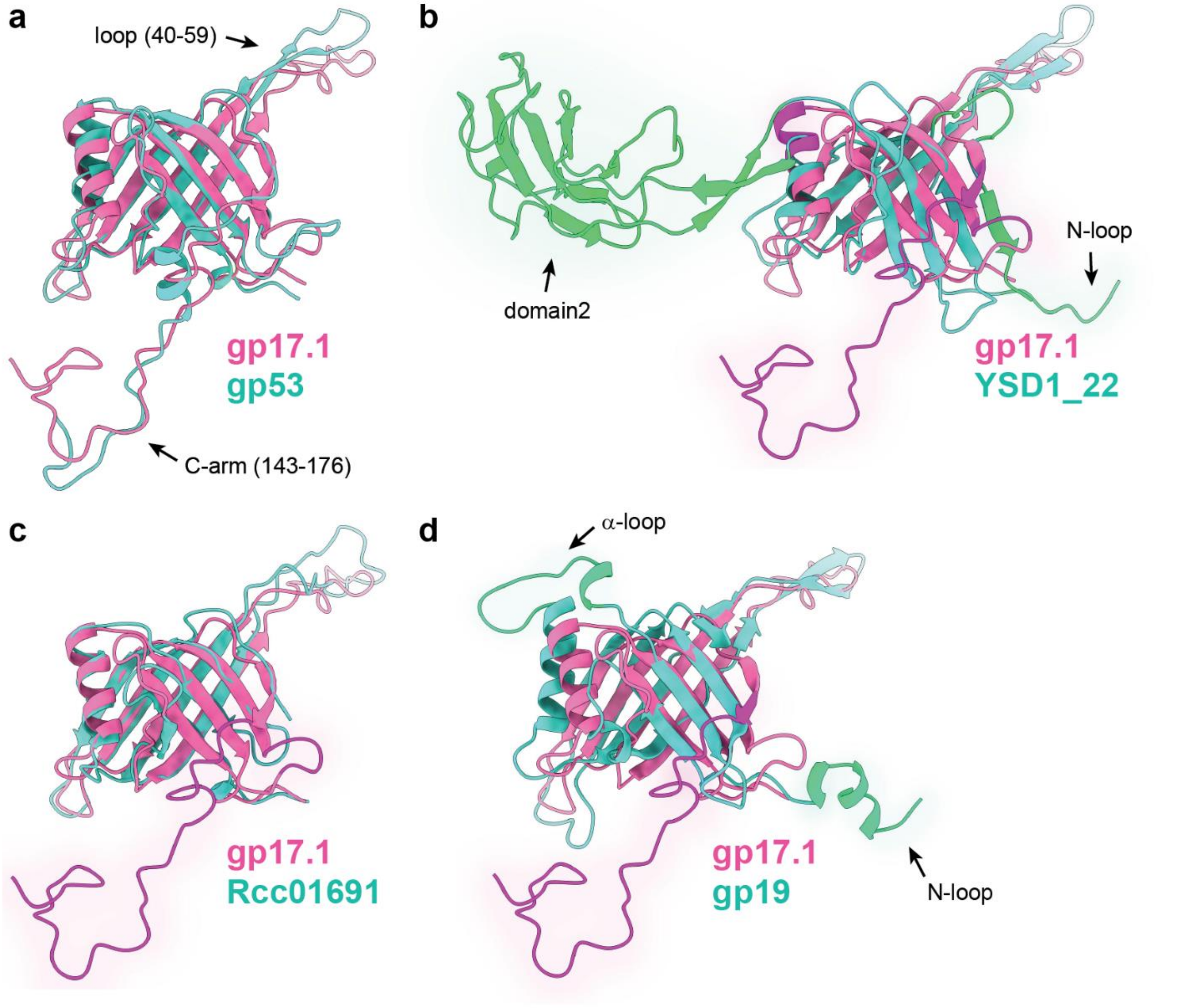

Figure S7. Structural alignment of the TTPs gp17.1 (*Siphoviridae*, SPP1 phage), and gp53[3] (*Siphoviridae*, 80α phage), YSD1_22[4] (*Siphoviridae*, YSD1), Rcc01691[5] (*Rhodobacter capsulatus*, gene transfer agent) and gp19[6] (*Myoviridae*, T4 phage) disregarding additional Ig-like domains. Structural alignments were performed with ChimeraX using the Needleman-Wunsch algorithm with a BLOSUM-62 residue similarity matrix of weight 0.7 and secondary structure scoring of weight 0.3.[7] In general, all TTPs share a common fold with RMSDs of 0.97 Å for gp17.1 and gp53 (**a**, between 67 pruned CA atom pairs), 1.16 Å for gp17.1 and YSD1_22 (**b**, between 11 pruned CA atom pairs), 1.08 Å for gp17.1 and Rcc01691 (**c**, between 35 pruned CA atom pairs), and 1.36 Å for gp17.1 and gp19 (**d**, between 21 pruned CA atom pairs). Structural differences are highlighted in magenta for gp17.1 and in green for the others. All TTPs share a common fold consisting of a β-sandwich-type fold that is flanked by an α-helix. gp17.1 and gp53 additionally feature similar loop regions – the C-arm (143-176) (even if not completely resolved in gp53) and the loop (40-59). YSD1_22 features an additional N-terminal loop (N-loop) and a domain (domain2) that embraces the neighboring subunit but lacks the ring-ring connecting C-terminal extension. Rcc01691 has no additional loops and lacks the ring-ring connecting C-terminal extension. gp19 lacks the ring-ring connecting C-terminal extension but has two additional loop regions (N-loop, α-loop).

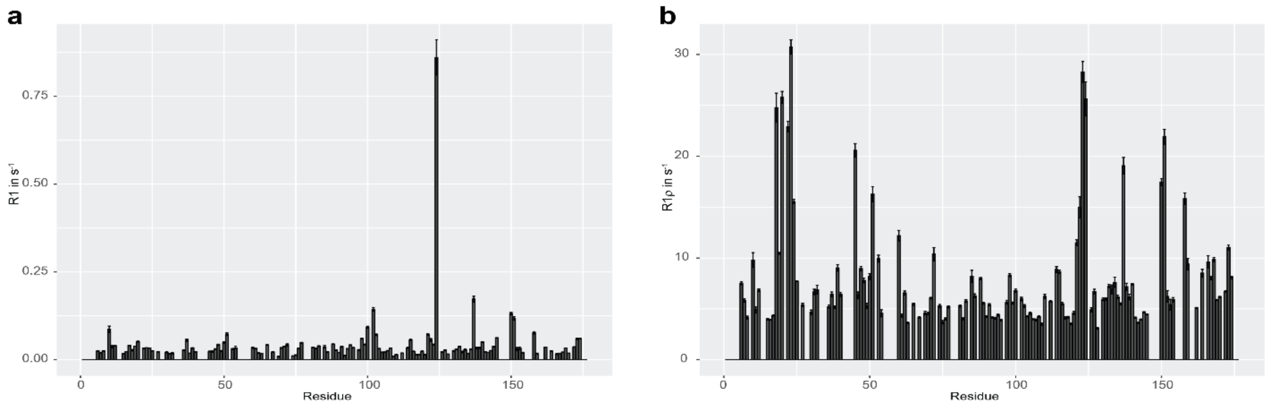

Figure S8. Relaxation rates a) R_1 and b) $R_{1\rho}$ as a function of residue number. Missing values represent residues that superimpose in the 2D hNH spectrum. The data were collected at 40 kHz MAS, 900 MHz external magnetic field strength and a temperature of +18 °C. The $R_{1\rho}$ rates were collected with a spin lock field of 5 kHz. The error bars represent twice the standard deviation of the fitting errors, which were estimated from Monte Carlo simulations with 1000 repetitions using the spectral noise level as input. Source data are provided as a Source Data file.

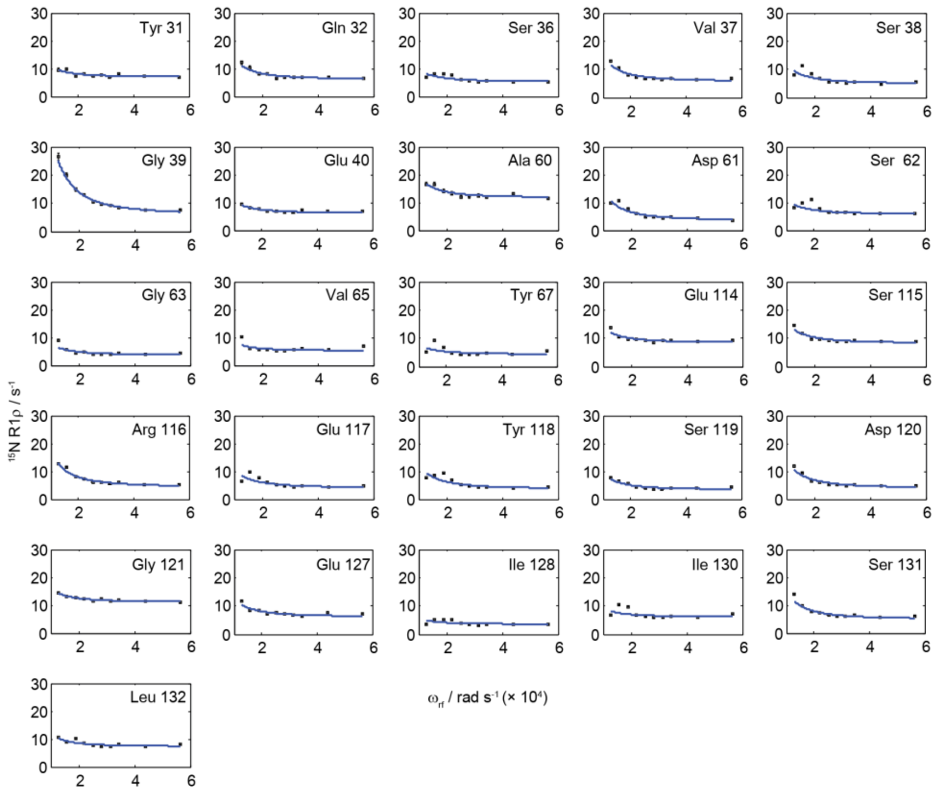

Figure S9. Combined fit of ^{15}N R1ρ relaxation dispersion plots of residues belonging to the inner β-barrel to a two-state Bloch McConell exchange process. The fit was conducted with a global exchange coefficient k_{ex}, individual ϕ_{ex} and individual R1ρ rates. The data were collected at 40 kHz MAS, 900 MHz external magnetic field strength and a temperature of +18 °C. Source data are provided as a Source Data file.

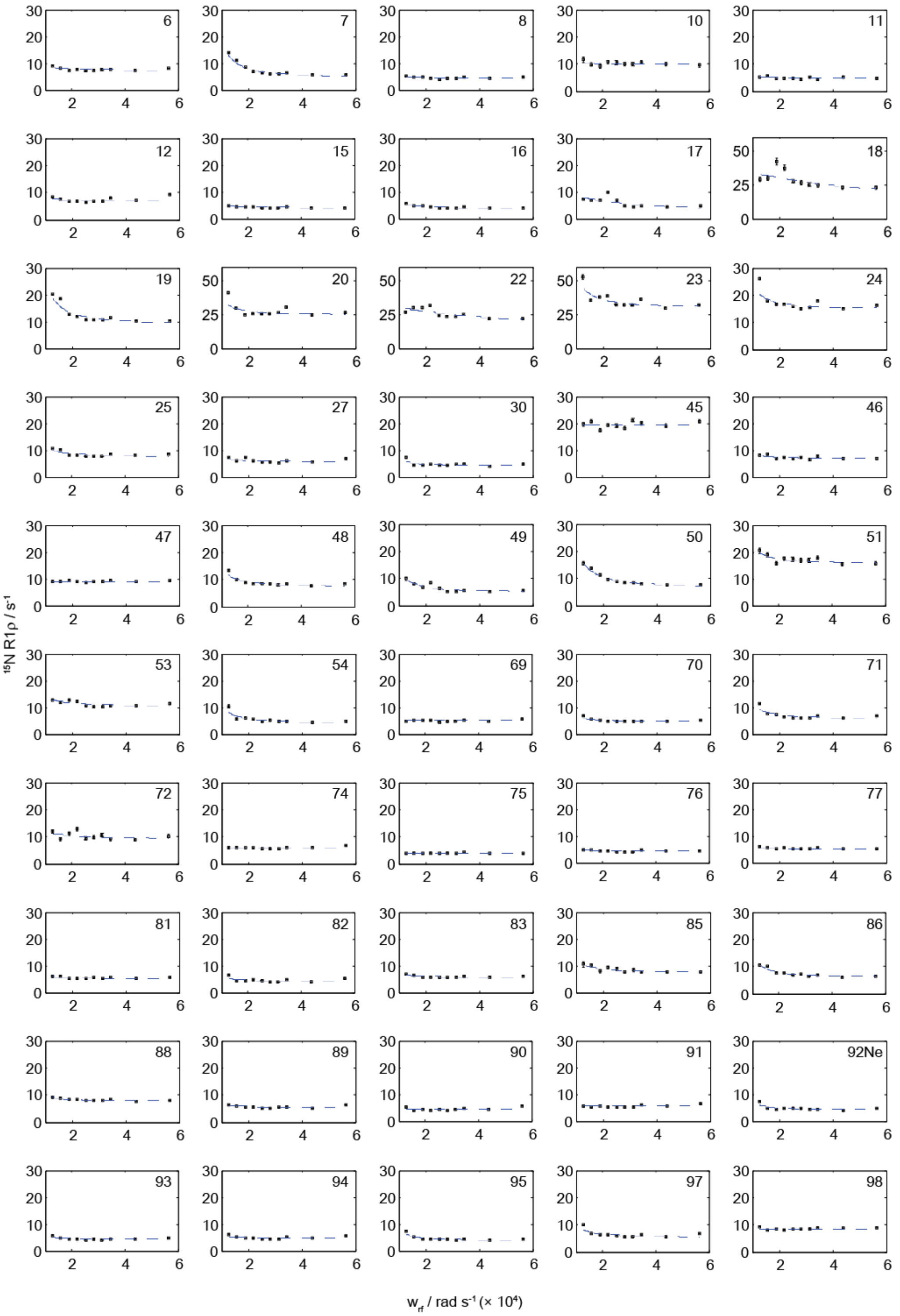

Figure S10. ^{15}N R1ρ relaxation dispersion plots of residues 6 to 98 of gp17.1. The line represents the best fit to a two-state Bloch McConell equation as described in the Methods. Missing residues superimpose in the 2D hNH spectrum and/or are unassigned. The data were collected at 40 kHz MAS, 900 MHz external magnetic field strength and a temperature of +18 °C. Ile18, Ala20, Thr22 and Gly23 have a different y-axis scaling. Source data are provided as a Source Data file.

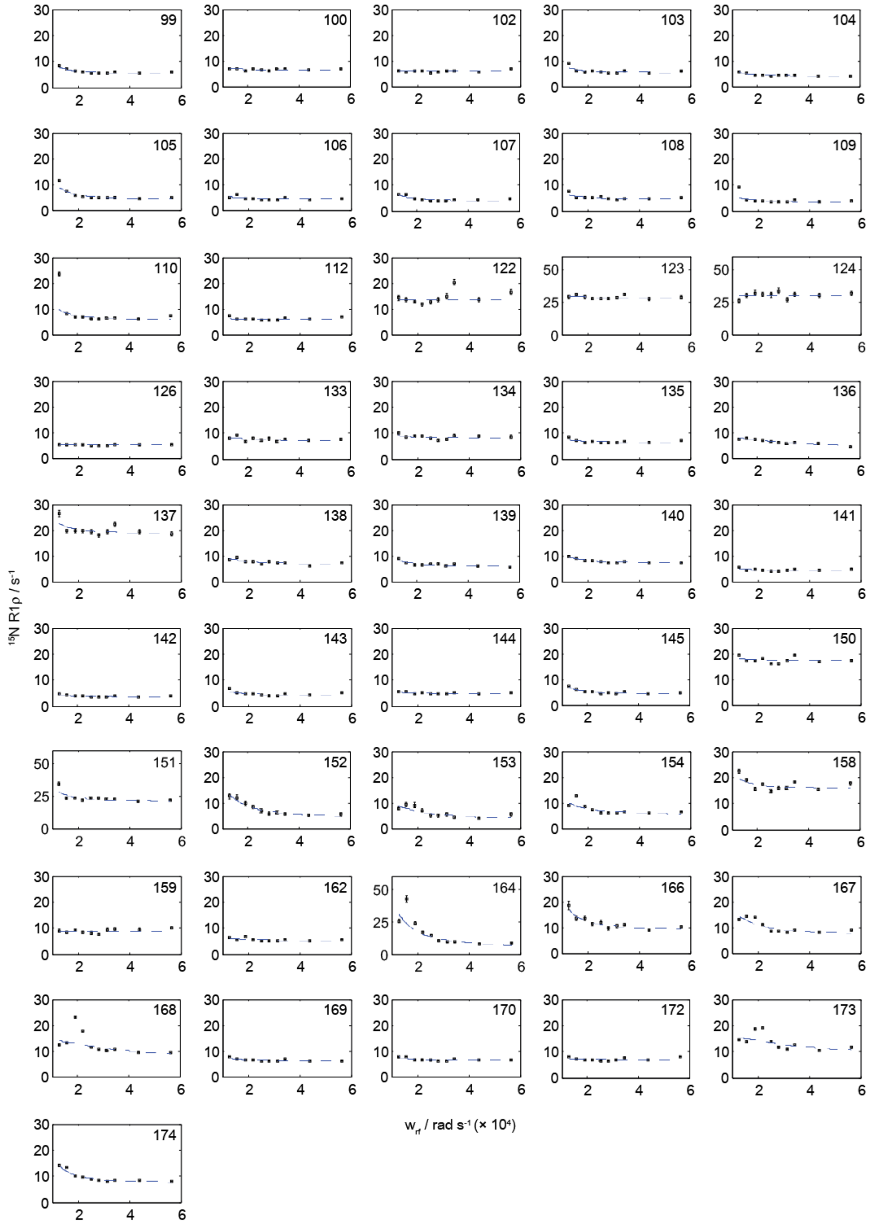

Figure S11. ^{15}N R1ρ relaxation dispersion plots of residues 99 to 174 of gp17.1. The line represents the best fit to a two-state Bloch McConell equation as described in the Methods. Missing residues superimpose in the 2D hNH spectrum and/or are unassigned. The data were collected at 40 kHz MAS, 900 MHz external magnetic field strength and a temperature of +18 °C. Glu123, Gly124, Val151 and Gly164 have a different y-axis scaling. Source data are provided as a Source Data file.

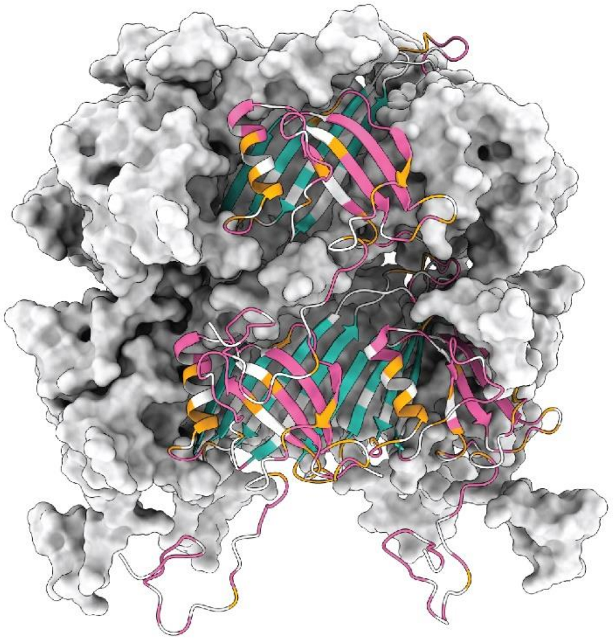

Figure S12. ^{15}N relaxation dispersion mapped onto three subunits of two rings of the SPP1 tail tube. Residues highlighted in pink and turquoise show non-flat relaxation dispersion profiles and are, thus, involved in motions on the millisecond timescale. Residues turquoise can be fitted in a combined manner to a two-state Bloch McConell exchange process. Residues in orange show flat profiles, residues in white superimpose or are unassigned. The direction of the tail structure is baseplate upwards. Source data are provided as a Source Data file.

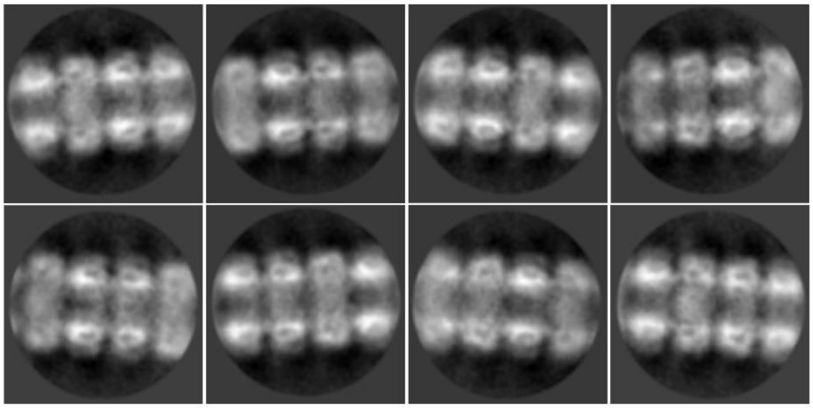

Figure S13. Exemplary 2D class averages for the straight segments.

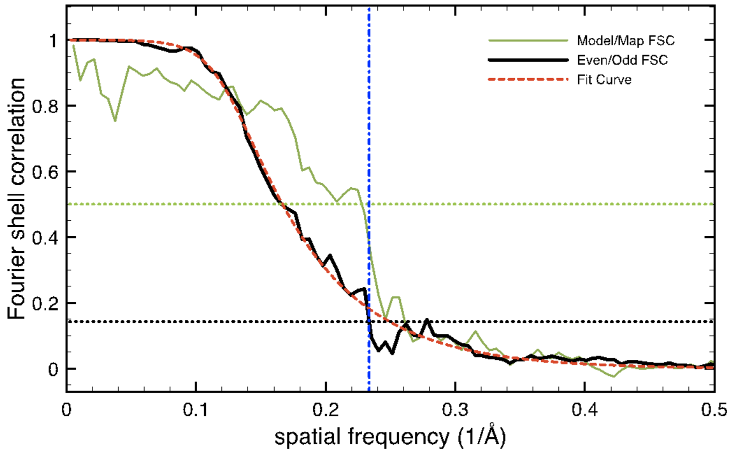

Figure S14. Fourier shell correlation (FSC) calculated between two half maps. According to the 0.143 criterion the obtained resolution is 4.3 Å. The FSC curve was fitted using $1/[e^{((x-A)/B)}+1]^{C}$, yielding A=0.122, B=0.015, and C=0.228. The fit yields a more robust resolution estimate of 4.0 Å (red). The model/map FSC (green) yields a similar cross-resolution estimate (at FSC of 0.5).

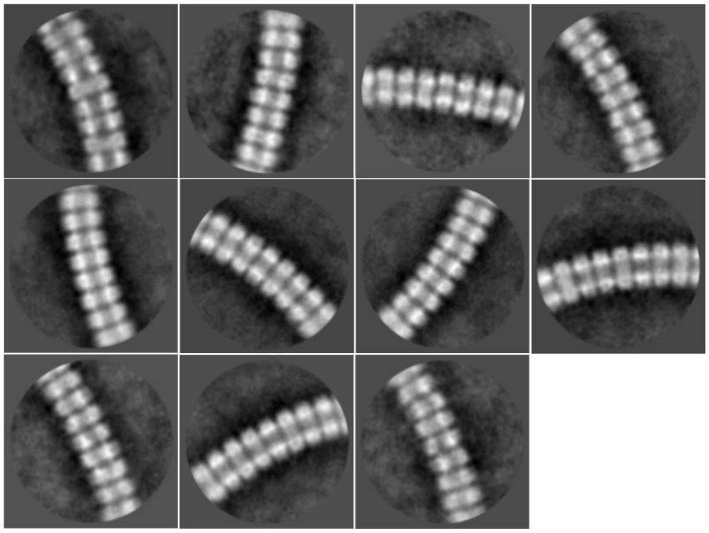

Figure S15. 2D class averages of bent tail tubes.

Table S1. Methyl-labeled and/or deuterated protein samples used in this study. The precursors were supplemented to the bacterial culture as described by us in Zinke et al. 2018 and in the protocols provided by NMR-Bio (Grenoble).

Labeling scheme	Labeling alias	Methyl-precursor
u-(^{2}H, ^{13}C, ^{15}N)	uniform	n/a
isoleucine-(^{1}Hδ1, ^{13}Cδ1)-u-^{15}N	isoleucine-methyl	2-Ketobutyric acid-4-^{13}C-3,3-d$_2$ (Sigma Aldrich)[8]
50% isoleucine-(^{1}Hδ1, ^{13}Cδ1)-u-^{14}N & 50% u-^{15}N	isoleucine-methyl mix	2-Ketobutyric acid-4-^{13}C-3,3-d$_2$ (Sigma Aldrich)[8]
alanine-(^{1}Hβ, ^{13}Cβ)-u-^{15}N	alanine-methyl	SLAM-A^{β} Kit from NMR-Bio[9]
50% alanine-(^{1}Hβ, ^{13}Cβ)-u-^{14}N & 50% u-^{15}N	alanine-methyl mix	SLAM-A^{β} Kit from NMR-Bio[9]
leucine-(^{1}Hδ, ^{13}Cδ)^{proR}-valine-(^{1}Hγ, ^{13}Cγ)^{proR}-u-^{15}N	LV-methyl	DLAM-LV^{proR} Kit from NMR-Bio[10]
50% leucine-(^{1}Hδ, ^{13}Cδ)^{proR}- valine-(^{1}Hγ, ^{13}Cγ)^{proR}-u-^{14}N & 50% u-^{15}N	LV-methyl mix	DLAM-LV^{proR} Kit from NMR-Bio[10]
threonine-(^{1}Hγ2, ^{13}Cγ2)-u-^{15}N	threonine-methyl	SLAM-T Kit from NMR-Bio[11]
50% threonine-(^{1}Hγ2, ^{13}Cγ2)-u-^{14}N & 50% u-^{15}N	threonine-methyl mix	SLAM-T Kit from NMR-Bio[11]
methionine-(^{1}Hε, ^{13}Cε)-u-^{15}N	methionine methyl	SLAM-M^{ε} Kit from NMR-Bio[12]
alanine-(^{1}Hβ, ^{13}Cβ)-isoleucine-(^{1}Hδ1, ^{13}Cδ1)-(^{1}Hγ2, ^{13}Cγ2)-leucine-(^{1}Hδ, ^{13}Cδ)$^{proR/proS}$-valine-(^{1}Hγ, ^{13}Cγ)$^{proR/proS}$- u-^{15}N	assignment	QLAM-A^{β}I$^{δ1/γ2}$LV$^{proR/proS}$ Kit from NMR-Bio[13]

Table S2. Summary of the acquired solid-state NMR spectra including their purpose. Methyl-labeled and/or deuterated samples were studied at 40 kHz MAS. The fully-protonated samples were studied at 11 kHz MAS.

			Summary of NMR spectra used in this study	
**Spinning Speed (rotor type)**	**Protein Sequence**	**Experiments**	**Sample**	**Purpose**
11 kHz (3.2 mm)	gp17.1	2D hCC	fully-protonated	fingerprint
	ΔN-3 gp17.1	2D hCC	fully-protonated	fingerprint
40 kHz (1.9 mm)	gp17.1	2D hCH	isoleucine-methyl	fingerprint
			alanine-methyl	
			LV-methyl	
			threonine-methyl	
			methionine methyl	
			assignment	
		3D HNhH	isoleucine-methyl	long-distance restraints
			alanine-methyl	
			LV-methyl	
			threonine-methyl	
			methionine methyl	
			alanine-methyl mix	intermolecular long-distance restraints
			isoleucine-methyl mix	
			LV-methyl mix	
			threonine-methyl mix	
		3D HChH	isoleucine-methyl	long-distance restraints
			LV-methyl	
		4D HNhhNH	uniform	long-distance restraints
		pseudo-3D	uniform	relaxation rates

Table S3. Parameters used for the spectral reconstruction of the non-uniformly sampled 4D spectrum. The reconstruction was conducted using the hmsIST software package.[14]

	Complex points after reconstruction				
**Experiment**	**F1**	**F2**	**F3**	**Iterations**	**Level Multiplier**
4D HNhhNH	48	30	48	3000	0.995

Table S4. Pulse program parameters. All experiments were conducted at a magic-angle spinning rate of 40 kHz and an external B_0 field corresponding to 900 MHz ^{1}H Larmor frequency. Unless mentioned otherwise, carrier positions were set to the center of the chemical shift range.

**Parameter**	**Value**			
**Experiment**	**2D hCH**	**3D HNhH**	**3D HChH**	**4D HNhhNH**
**Samples**				
Labeling alias	isoleucine-methyl<br>alanine-methyl<br>LV-methyl<br>threonine-methyl<br>methionine methyl	isoleucine-methyl<br>isoleucine-methyl mix<br>alanine-methyl<br>alanine-methyl mix<br>LV-methyl<br>LV-methyl mix<br>threonine-methyl<br>threonine-methyl mix<br>methionine methyl	isoleucine-methyl<br>LV-methyl	uniform
**Recycle delay**				
Recycle delay	1 s	1.15 s	1 s	1 s
**90° initial ^{1}H excitation pulse**				
R.f. power	100 kHz	100 kHz	100 kHz	83.3 kHz
Duration	2.5 µs	2.5 µs	2.5 µs	3 µs
Carrier position		8.5 ppm		8.5 ppm
**^{1}H evolution time**				
WALTZ r.f. power		3.4 kHz (^{15}N)	4.9 kHz (^{13}C)	7.9 kHz (^{15}N)
WALTZ pulse duration		60 µs	60 µs	60 µs
WALTZ carrier position		117.7 ppm		117.7 ppm
**^{1}H-^{15}N CP step**				
^{1}H r.f. power		81 kHz		78.7 kHz
^{1}H carrier position		8.5 ppm		8.5 ppm
^{15}N r.f. power		29.6 kHz		30.4 kHz
^{15}N carrier position		117.7 ppm		117.7 ppm
Ramp shape		Ramp 80-100% on ^{1}H		Ramp 80-100% on ^{1}H
Duration		1400 µs		900 µs
**^{15}N evolution time**				
WALTZ r.f. power		9.5 kHz (^{1}H)		8.5 kHz (^{1}H)
WALTZ pulse duration		40 µs		50 µs
WALTZ carrier position		8.5 ppm		8.5 ppm
**^{1}H-^{13}C CP step**				
^{1}H r.f. power	52.5 kHz		54.0 kHz	
^{1}H carrier position				
^{13}C r.f. power	10.2 kHz		10.2 kHz	
^{13}C carrier position				
Ramp shape	Ramp 80-100% on ^{1}H		Ramp 80-100% on ^{1}H	
Duration	2 ms		1.7 ms	
**^{13}C evolution time**				
WALTZ r.f. power	2.8 kHz (^{1}H)		3.6 kHz (^{1}H)	
WALTZ pulse duration	40 µs		40 µs	
WALTZ carrier position				
**90° ^{15}N/^{13}C flip pulses**				
R.f. power	50 kHz	35.7 kHz	50 kHz	35.7 kHz
Duration	5 µs	7 µs	5 µs	7 µs
Carrier position		117.7 ppm		117.7 ppm
**Water suppression**				
T delay	44 ms	44 ms	44 ms	42 ms
Spoil pulse r.f. power	43.6 kHz	43.6 kHz	43.6 kHz	
Spoil pulse duration	1 ms	1 ms	1 ms	
Spoil pulse shape	Ramp 100-60%	Ramp 100-60%	Ramp 100-60%	
First pulse duration in train	33 ms	33 ms	33 ms	38 ms
Second pulse duration in train	56 ms	56 ms	56 ms	64.4 ms
Train r.f. power	13.8 kHz	13.8 kHz	13.8 kHz	16.4 kHz

Wat. sup. carrier position	4.9 ppm	4.9 ppm	4.9 ppm	4.9 ppm
Loops through train (n)	1	1	1	1
Gradient pulse shape				SINE.100
Gradient pulse power				100%
Gradient pulse duration				1 ms
Delay for ring down				500 µs
**^{15}N-^{1}H CP step**				
^{1}H r.f. power		81.6 kHz		78.7 kHz
^{1}H carrier position		8.5 ppm		8.5 ppm
^{15}N r.f. power		33.5 kHz		31.1 kHz
^{15}N carrier position		117.7 ppm		117.7 ppm
Ramp shape		Ramp 100-80% on ^{1}H		Ramp 100-80% on ^{1}H
Duration		900 µs		800 µs
**^{13}C-^{1}H CP step**				
^{1}H r.f. power	52.5 kHz		51.6 kHz	
^{1}H carrier position				
^{13}C r.f. power	10.2 kHz		10.2 kHz	
^{13}C carrier position				
Ramp shape	Ramp 100-80% on ^{1}H		Ramp 100-80% on ^{1}H	
Duration	2 ms		900 µs	
**^{1}H-^{1}H RFDR Mixing**				
RFDR r.f. power		100 kHz	100 kHz	100 kHz
RFDR mixing time		10 ms	6 ms	6 ms
**2nd ^{1}H-^{15}N CP step**				
^{1}H r.f. power				78.7 kHz
^{1}H carrier position				8.5 ppm
^{15}N r.f. power				31.1 kHz
^{15}N carrier position				117.7 ppm
Ramp shape				Ramp 80-100% on ^{1}H
Duration				800 µs
**Acquisition**				
^{15}N WALTZ r.f. power		3.4 kHz		7.9 kHz
^{15}N WALTZ pulse duration		60 µs		60 µs
^{15}N WALTZ carrier position		117.7 ppm		117.7 ppm
^{13}C WALTZ r.f. power	9.8 kHz	5 kHz	4.9 kHz	
^{13}C WALTZ pulse duration	60 µs	60 µs	60 µs	
^{13}C WALTZ carrier position		24.9 ppm		

Table S5. Acquisition parameters for 2D, 3D, and 4D spectra. The highest dimension is always the direct dimension. Parameters for 2D hCH, 3D HNhH, and 3D HChH spectra are exemplarily shown for the LV-methyl labeled species. Acquired points for further methyl-labeled samples vary due to inherently different chemical shift dispersions. The 4D HNhhNH spectrum was recorded on the uniformly labeled sample and with non-uniform sampling (25 %).

	Acquisition time / ms (number of complex points)						
**Experiment**	**F1**	**F2**	**F3**	**F4**	**ns**	**Total Number of acquired points**	**Total Time**
2D hCH (LV-methyl)	15.2 ms (38) (^{13}C)	20 ms (512) (^{1}H)	N/A	N/A	48	76	1 h 23 min
3D HNhH (LV-methyl)	7.5 ms (30) (^{1}H)	15 ms (45) (^{15}N)	20 ms (512) (^{1}H)	N/A	16	5400	1 d 15 h 34 min
3D HChH (LV-methyl)	7 ms (14) (^{1}H)	10 ms (21) (^{13}C)	20 ms (512) (^{1}H)	N/A	32	1176	15 h 41 min
4D HNhhNH	10.6 ms (32) (^{15}N)	6.2 ms (23) (^{1}H)	10.6 ms (32) (^{15}N)	21.3 ms (512) (^{1}H)	8	47448/188416	7 d 2 h 28 min

Table S6. Processing parameters for 2D, 3D and 4D spectra. The highest dimension is always the direct dimension.

	Points after FT				Window function			
**Experiment**	**F1**	**F2**	**F3**	**F4**	**F1**	**F2**	**F3**	**F4**
2D hCH	1k (^{13}C)	4k (^{1}H)	N/A	N/A	sin^2, φ=45°	sin^2, φ=45°	N/A	N/A
3D HNhH	128 (^{1}H)	128 (^{15}N)	4k (^{1}H)	N/A	sin^2, φ=60°	sin^2, φ=60°	sin^2, φ=60°	N/A
3D HChH	128 (^{1}H)	128 (^{13}C)	2k (^{1}H)	N/A	sin^2, φ=60°	sin^2, φ=60°	sin^2, φ=60°	N/A
4D HNhhNH	64 (^{15}N)	64 (^{1}H)	64 (^{15}N)	1k (^{1}H)	sin^2, φ=90°	sin^2, φ=90°	sin^2, φ=90°	sin^2, φ=60°

Table S7. Values for the fitting of the relaxation dispersion profiles of the inner β-barrel residues to a two-state Bloch McConell exchange process. Exemplary chemical shift differences between the two sites p_A (95%) and p_B (5%) are extracted from the ϕ_{ex} individual values.

Residue	Global exchange coefficient k_{ex} (s^{-1})	Individual ϕ_{ex} (10^3 rad^2 s^{-2})	Individual $R_{1\rho}$ (s^{-1})	Chemical shift differences (ppm)
31	4500 ± 2300	106 ± 90	6.97 ± 0.39	2.64 ± 2.23
32		200 ± 200	6.10 ± 0.66	3.65 ± 3.61
36		120 ± 94	5.28 ± 0.20	2.81 ± 2.19
37		240 ± 190	5.75 ± 0.40	3.93 ± 3.23
38		180 ± 140	4.95 ± 0.21	3.48 ± 2.66
39		760 ± 630	5.90 ± 0.59	7.07 ± 5.83
40		118 ± 84	6.18 ± 0.27	2.78 ± 1.98
60		200 ± 170	11.67 ± 0.78	3.65 ± 3.11
61		290 ± 230	3.51 ± 0.27	4.35 ± 3.54
62		141 ± 95	5.79 ± 0.28	3.05 ± 2.03
63		111 ± 89	3.69 ± 0.13	2.70 ± 2.17
65		81 ± 61	5.21 ± 0.14	2.31 ± 1.72
67		93 ± 65	4.29 ± 0.12	2.47 ± 1.72
114		150 ± 120	8.43 ± 0.52	3.11 ± 2.57
115		196 ± 15	8.26 ± 0.24	3.59 ± 2.91
116		340 ± 260	4.37 ± 0.22	4.70 ± 3.57
117		180 ± 130	4.11 ± 0.19	3.44 ± 2.55
118		240 ± 160	3.74 ± 0.22	3.93 ± 2.60
119		150 ± 130	3.41 ± 0.19	3.18 ± 2.72
120		270 ± 210	4.02 ± 0.24	4.20 ± 3.24
121		130 ± 110	11,11 ± 0.39	2.94 ± 2.41
127		180 ± 120	6.19 ± 0.48	3.41 ± 2.27
128		53 ± 44	3.55 ± 0.14	1.86 ± 1.54
130		82 ± 60	6.13 ± 0.24	2.32 ± 1.70
131		270 ± 200	5.02 ± 0.22	4.17 ± 3.19
132		120 ± 100	7.30 ± 0.23	2.79 ± 2.38

Table S8. Cryo-EM data collection, refinement and validation statistics

	EMD-10792, PDB ID 6YEG
**Data collection and processing**	
Magnification	110,000
Voltage (kV)	200
Total dose (e^{-}/Å^2)	70
Exposure time (s)	3
Movie frames (no.)	120
Defocus range (μm)	-0.2 to -1.7
Pixel size (Å)	0.935
No. Micrographs	855
Symmetry imposed	C6, helical
Helical rise (Å)	38.46
Helical twist (º)	21.89
Final fibril images (no.)	1866
Final particle images (no.)	5965
Map resolution (Å)	4.3
FSC threshold	0.143
**Refinement**	
Initial density model used	sphere model based on estimated helical symmetry
Model composition	
Non-hydrogen atoms	15768
Protein residues	2064
Chains	12
R.m.s. deviations	
Bond lengths (Å)	0.0063
Bond angles (°)	1.32
**Validation**	
MolProbity score	2.24
Clashscore	11.2
Poor rotamers (%)	0.8
Ramachandran plot	
Favored (%)	84.4
Disallowed (%)	0.3

Table S9. Overview of long-range distance restraints from solid-state NMR. The numbers of spectrally unambiguous (chemical shift cutoffs ^{15}N ~0.15 ppm, ^{13}C ~0.15 ppm, ^{1}HN ~0.05 ppm, ^{1}Hmethyl ~0.03 ppm) restraints are shown in brackets.

**Categories**	**Sequence separation**
intra-residue	0
sequential	1
medium-range	2-4
long-range	>4

**NMR inter (only between subunits)**	**Number**
intra-residue	0
sequential	0
medium-range	0
long-range	17
total (spectrally unambiguous)	17 (9)

**NMR ambiguous (within and between subunits)**	**Number**
intra-residue	12
sequential	120
medium-range	75
long-range	480
total (spectrally unambiguous)	687 (406)

**Hydrogen bonds (within subunit)**	**Number**
intra-residue	0
sequential	0
medium-range	16
long-range	80

**Hydrogen bonds (between subunits)**	**Number**
intra-residue	0
sequential	0
medium-range	0
long-range	12

Table S10. Violations of solid-state NMR long-range distance restraints.

NMR inter (only between subunits)	Number
total number of restraints	17
violations 7.0 – 8.0 Å	1
violations 8.0 – 9.0 Å	1
violations 9.0 – 10.0 Å	0
violations > 10 Å	0

NMR ambiguous (within and between subunits)	Number
total number of restraints	687
violations 7.0 – 8.0 Å	63
violations 8.0 – 9.0 Å	35
violations 9.0 – 10.0 Å	18
violations > 10 Å	9

Table S11. Ensemble RMSDs after hybrid structure calculation (PDB ID 6YQ5). For each pair from the ensemble the RMSD between coordinates of an atom selection was computed using the Kabsch algorithm. These values were then averaged over all combinations and the standard deviation of these was computed. There is significant difference between the RMSDs within a single subunit and the corresponding RMSD values computed for the entire assembly.

Subunit	RMSD
C-alpha atoms	1.1 ± 0.5 Å
Backbone atoms	1.1 ± 0.5 Å
Heavy atoms	1.8 ± 0.9 Å

Full assembly (12-mer)	RMSD
C-alpha atoms	1.1 ± 0.5 Å
Backbone atoms	1.1 ± 0.5 Å
Heavy atoms	1.8 ± 0.9 Å

Supporting References

1. Dolinsky, T. J., Nielsen, J. E., McCammon, J. A. & Baker, N. A. PDB2PQR: An automated pipeline for the setup of Poisson-Boltzmann electrostatics calculations. *Nucleic Acids Res.* **32**, 665–667 (2004).

2. Baker, N. A., Sept, D., Joseph, S., Holst, M. J. & McCammon, J. A. Electrostatics of nanosystems: Application to microtubules and the ribosome. *Proc. Natl. Acad. Sci. U. S. A.* **98**, 10037–10041 (2001).

3. Kizziah, J. L., Manning, K. A., Dearborn, A. D. & Dokland, T. Structure of the host cell

recognition and penetration machinery of a Staphylococcus aureus bacteriophage. *PLoS Pathog.* **16**, e1008314 (2020).

4. Hardy, J. M. *et al.* The architecture and stabilisation of flagellotropic tailed bacteriophages. *Nat. Commun.* **11**, 3748 (2020).

5. Bárdy, P. *et al.* Structure and mechanism of DNA delivery of a gene transfer agent. *Nat. Commun.* **11**, 3034 (2020).

6. Zheng, W. *et al.* Refined Cryo-EM Structure of the T4 Tail Tube: Exploring the Lowest Dose Limit. *Structure* **25**, 1436-1441.e2 (2017).

7. Goddard, T. D. *et al.* UCSF ChimeraX: Meeting modern challenges in visualization and analysis. *Protein Sci.* **27**, 14–25 (2018).

8. Gardner, K. H. & Kay, L. E. Production and incorporation of 15N, 13C, 2H (1H-δ1 methyl) isoleucine into proteins for multidimensional NMR studies. *J. Am. Chem. Soc.* **119**, 7599–7600 (1997).

9. Ayala, I., Sounier, R., Usé, N., Gans, P. & Boisbouvier, J. An efficient protocol for the complete incorporation of methyl-protonated alanine in perdeuterated protein. *J. Biomol. NMR* **43**, 111–119 (2009).

10. Gans, P. *et al.* Stereospecific isotopic labeling of methyl groups for NMR spectroscopic studies of high-molecular-weight proteins. *Angew. Chemie - Int. Ed.* **49**, 1958–1962 (2010).

11. Velyvis, A., Ruschak, A. M. & Kay, L. E. An Economical Method for Production of 2H,13CH3-Threonine for Solution NMR Studies of Large Protein Complexes: Application to the 670 kDa Proteasome. *PLoS One* **7**, 1–8 (2012).

12. Fischer, M. *et al.* Synthesis of a13C-methyl-group-labeled methionine precursor as a

useful tool for simplifying protein structural analysis by NMR spectroscopy. *ChemBioChem* **8**, 610–612 (2007).

13. Kerfah, R., Hamelin, O., Boisbouvier, J. & Marion, D. CH3-specific NMR assignment of alanine, isoleucine, leucine and valine methyl groups in high molecular weight proteins using a single sample. *J. Biomol. NMR* **63**, 389–402 (2015).

14. Hyberts, S. G., Milbradt, A. G., Wagner, A. B., Arthanari, H. & Wagner, G. Application of iterative soft thresholding for fast reconstruction of NMR data non-uniformly sampled with multidimensional Poisson Gap scheduling. *J. Biomol. NMR* **52**, 315–327 (2012).
