# Supplementary material for: Architecture of the flexible tail tube of bacteriophage SPP1
Source: Nat Commun. 2020 Nov 13;11:5759. doi: 10.1038/s41467-020-19611-1 (PMC7666168; doi:10.1038/s41467-020-19611-1)
Supplement: Supplementary file 2 — Description of Additional Supplementary Files [file 41467_2020_19611_MOESM2_ESM.pdf]

Description of Additional Supplementary Files

File Name: Supplementary Movie 1

Description: Overall structure of the flexible tail tube of bacteriophage SPP1. One subunit is highlighted in pink. Subsequently, its secondary structure elements are rainbow-coloured. The loop (40-59) and the C-arm (143-176) are displayed. The hexameric architecture of the tail tube is emphasised by highlighting certain subunits in pink and turquoise. The solid-state NMR restraints and cryo-EM map for the hybrid structure calculation are visualized. The inner [illegible]-barrel that forms the lumen of the tube is visualized.

File Name: Supplementary Movie 2

Description: Bending of the flexible tail tube of bacteriophage SPP1. One subunit is highlighted in pink. The bending process is mediated by stretching of certain linker regions as opposed to compression.
